# Dietary lipid overload creates a suppressive environment that impedes the antiviral functions of NK cells

**DOI:** 10.1016/j.isci.2025.112396

**Published:** 2025-04-09

**Authors:** Simone Schimmer, Leonie Kerkmann, Nele Kahlert, Shahd al Jubeh, Tanja Werner, Carrie Corkish, Hannah Prendeville, David K. Finlay, Kathrin Sutter, Ulf Dittmer, Elisabeth Littwitz-Salomon

**Affiliations:** 1 Institute for Virology, University Hospital Essen, University of Duisburg-Essen, Essen, Germany; 2 Institute for the Research on HIV and AIDS-associated Diseases, University Hospital Essen, University of Duisburg-Essen, Essen, Germany; 3 School of Biochemistry and Immunology, Trinity Biomedical Sciences Institute, Trinity College Dublin, 152-160 Pearse Street, Dublin 2, Ireland; 4 Biomedical Engineering, School of Engineering, College of Science and Engineering, University of Galway, Galway, Ireland; 5 School of Pharmacy and Pharmaceutical Sciences, Trinity Biomedical Sciences Institute, Trinity College Dublin, 152-160 Pearse Street, Dublin 2, Ireland

**Keywords:** Natural killer cells, obesity, regulatory T cells, Friend retrovirus, Interleukin-10, suppressive microenvironment

## Abstract

Natural killer (NK) cells are innate immune cells able to recognize and eliminate virus-infected cells. NK cell activity strongly correlates with a metabolic reprogramming and breakdown of fatty acids by β-oxidation during virus infections. However, there is limited knowledge regarding the impact of obesity on antiviral NK cell functions. Here, employing the Friend retrovirus mouse model, we show that the cytotoxicity and cytokine production of NK cells was impaired in obesity, leading to higher viral loads. NK cells suppression in obesity was mediated by activated Tregs. Furthermore, obese mice that were switched back to a regular diet showed complete recovery of the NK cell activity. Interestingly, feeding mice with a high-fat diet (HFD) for just ten days caused NK cell dysfunction and increased retroviral burden. This study is the first to link the detrimental impact of an obesity-induced immunosuppressive microenvironment with NK cell dysfunction during an acute retroviral infection.

## Highlights

• Dysfunctional NK cells in obesity and short-term high-fat diet (HFD)
• Reduction of body weight reinvigorated NK cell functions
• Tregs suppress NK cells in HFD during acute retrovirus infection
• IL-10 mediates the suppression of NK cell cytotoxicity in acute retrovirus infection

## Graphical abstract

**Figure:**
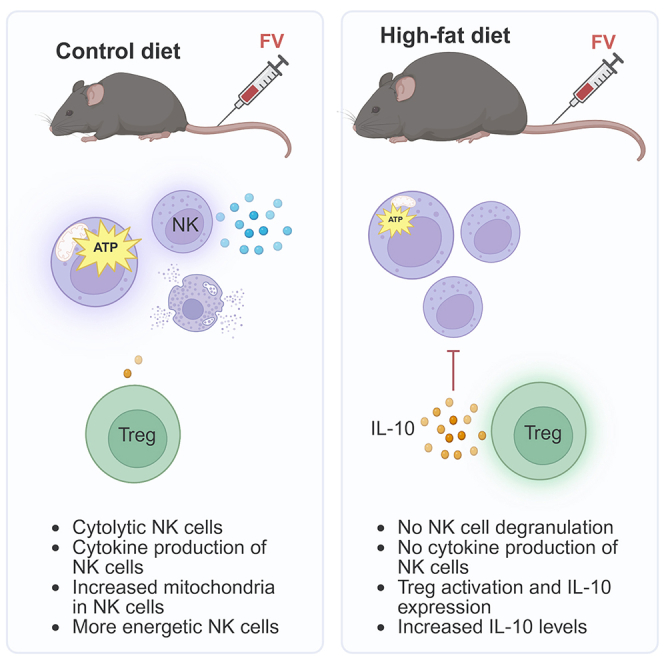

## Introduction

Natural killer (NK) cells are innate immune cells able to eliminate virus-infected cells as well as cancer cells. Considering the importance of NK cells in the immunity against retroviral infections such as human immunodeficiency virus (HIV), NK cells could be key players for the development of new strategies for retroviral cure. Induction of NK cell activation is mediated by germline-encoded activating or inhibitory receptors.^1^ The process begins with receptor-ligand interactions, such as NKG2D-RAE-1 and DNAM-1-CD155, which are crucial for recognizing infected cells.^2^ On encountering a susceptible target cell, cytotoxicity is facilitated by polarization and granule exocytosis into the immunological synapse.^3^ Granules contain several granzymes (Gzm) as well as perforin, which together induce apoptosis in susceptible target cells. Through the death receptor ligands, tumor necrosis factor related apoptosis inducing ligand (TRAIL) and Fas ligand (FasL), NK cells can further induce cell death in target cells that express the corresponding death receptors.^4^ Cytokines such as interferon (IFN)-γ and tumor necrosis factor (TNF) and chemokines like MIP-1α, MIP-1β, and RANTES are produced and released by NK cells, which can inhibit viral entry into target cells^5^ and regulate innate and adaptive immunity.^6^ Additionally, NK cells can engage in antibody-dependent cellular cytotoxicity (ADCC) through Fc receptor binding and contribute to immune control of virus infection.^7^ Simultaneously, cytokine signaling plays a vital role, with IL-15, IL-18, type I interferons, IL-2, and IL-12 stimulating NK cells and enhancing their antiviral functions.^8^^,^^9^ It has been shown that activated NK cells undergo metabolic reprogramming leading to cell proliferation, cytokine secretion, and the release of cytotoxic granules.^10^^,^^11^^,^^12^^,^^13^ Especially signaling through the transcription factor cMyc and the master regulator mammalian target of rapamycin (mTOR) controls metabolic rewiring in NK cells.^11^^,^^12^^,^^13^^,^^14^ cMyc transcriptionally regulates numerous genes encoding for mitochondrial proteins, thus, it is important for cell proliferation and growth.^15^ Proliferation requires energy, which is effectively produced in mitochondria through electron transfer and oxidative phosphorylation (OXPHOS) as well as cytosolic glycolysis. The uptake and availability of nutrients is important for the induction of NK cell activity.^13^ As demonstrated before, a nutrient-depleted tumor microenvironment resulted in dysfunctional NK cells.^16^ Interestingly, NK cells require the breakdown of fatty acids (β-oxidation) for energy production and activity during an acute retroviral infection.^14^ We have previously shown that NK cells increase the expression of the transporter CD36 during the acute retroviral infection, which is essential for the uptake of fatty acids. Besides retroviral infections, the importance of β-oxidation for NK cell cytotoxicity was also reported in mouse cytomegalovirus infection as well as some cancer entities.^17^ Free fatty acid levels are elevated in obesity and associated with insulin resistance. Obesity is a condition defined by excessive fat deposits that has been recognized as primary contributor to significant health issues, such as metabolic, cardiovascular, and oncological diseases.^18^^,^^19^ However, we and others previously reported that the lipid metabolism can be crucial for the activity of NK cells in acute viral infections.^14^^,^^17^ Thus, the role of cytotoxic NK cells in combating acute virus replication in the context of obesity is not completely understood so far. Here, we used the Friend retrovirus (FV) to analyze antiviral NK cell functions in diet-induced obese mice. FV is a simple gammaretrovirus with only *gag*, *pol*, and *env* genes compared with a complex retrovirus such as HIV.^20^ The FV complex consists of the replication-competent but apathogenic F-MuLV (Friend murine leukemia virus) and the SFFV (spleen focus-forming virus), which is replication-defective but pathogenic.^21^ Adult C57BL/6 mice are resistant to FV-induced disease and develop a strong immune response against the viral complex, which is comparable to HIV in humans in terms of Tregs,^22^^,^^23^ cytotoxic CD8^+^ T cell,^24^ and NK cell responses.^25^^,^^26^ However, they fail to completely clear the virus, which results in a lifelong chronic infection. The FV model is particularly crucial for investigating acute retroviral infections, which are rarely detected in humans due to the typically asymptomatic nature of early HIV infection.

Cytotoxicity of NK cells is indispensable to restrict viral spread in acute retroviral infection as it was shown for FV and HIV infection,^26^ and ablation of NK cells resulted in strongly increased viral burden during acute FV infection.^27^ NK cells are crucial in the acute phase of FV infection and control viral replication until cytotoxic CD8^+^ T cells become the primary mediators of anti-retroviral immunity.^27^^,^^28^ Activation of NK cells required the cytokines IL-15 and IL-18, but their function is strongly controlled to prevent severe immunopathology.^9^ One negative regulator of NK cell functions are suppressive T cells, called Tregs.^29^^,^^30^ NK cell activity depends on IL-2 and competing for IL-2 with Tregs resulted in reduced IL-2 availability for NK cells and diminished NK cell activation.^29^ Additionally, Tregs are able to produce the anti-inflammatory cytokines transforming growth factor (TGF), which has been shown to drive metabolic dysfunction in NK cells from patients with breast cancer,^31^ as well as IL-10.^32^ In FV infection, Tregs produce and release IL-10, which decreases the NK cell cytotoxicity.^30^ Similar to HIV infection, non-functional NK cells arise in chronic FV infection but similar to SIV infection in monkeys a small population of adaptive NK cells emerge in this phase of infection.^33^^,^^34^ Hence, FV infection serves as an excellent model to study NK cell responses against retroviruses in an obese host.

Fast activation, cytokine secretion, as well as cytotoxicity render NK cells an attractive target for immunotherapies in cancer and infectious diseases. However, little is known about NK cell function in obesity and the obese microenvironment, so potential therapeutic interventions might be missed. Using the FV infection model, we can analyze the initial NK cell responses against retroviral infection and assess the effects of dietary changes. Here, the antiviral cytotoxicity of NK cells was diminished in obesity, resulting in increased viral burden. Activated IL-10- producing Tregs strongly contribute to the suppressive microenvironment. Changing the diet from high-fat diet (HFD) to normal diet resulted in weight loss and reinvigorated NK cell functions. Obesogenic diet exposure for a short time period was sufficient to induce an impaired NK cell phenotype. This new understanding of NK cells and their suppressive environment in obesity is central for the development of new therapies in viral infections targeting the suppressive milieu as well as NK cells themselves.

## Results

### Impact of high-fat diet on mouse weight and FV-induced disease during acute retrovirus infection

**Figure 1:**
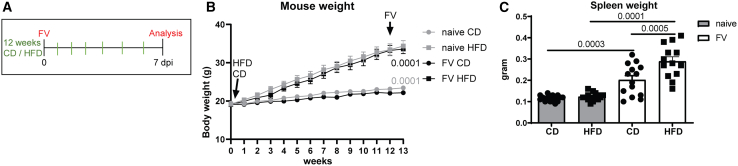
Increased mouse and spleen weight after feeding mice with HFD for three months C57BL/6 mice were fed with control diet (CD) or high-fat diet (HFD) for 13 weeks. After 12 weeks, mice were infected with FV or used as naive controls and analyzed at 7 days post infection (dpi). Overview of experimental setup is shown in (A). Mouse weight was measured weekly (B). Data were collected from four independent experiments. Statistically significant differences between CD (circles)- and HFD (squares)-fed mice of naive (gray) or FV-infected (black) mice were shown for body weights at week 13. At the day of experiments, spleens were weighed (C). In total, 15 mice (naive) and 14 mice (FV) per group from four independent experiments were used and analyzed by an unpaired t test (B, 13-week time point) and ordinary one-way ANOVA (C). The significance threshold was set at 0.05. Data are presented as mean values ± SEM.

C57BL/6 mice were fed with normal control diet (CD) or HFD for 13 weeks.^35^^,^^36^ Mice were infected with FV in week 12 or used as naive control mice (Figure 1A). Mice receiving HFD generated 45% of their calories from fat, which is physiologically relevant and comparable to a high-fat American or European human diet.^37^ Importantly, the control diet (10% energy by fat) contained purified ingredients, which were matched to nutritional composition of HFD and differ in the fat content. The weight of mice was monitored over 13 weeks (Figure 1B). Body weight of all mice increased over time. From week 2, weight gain was statistically significantly greater in mice fed the HFD compared to mice fed the CD (*p* = 0.0123). Mice exposed to high-fat chow reached more than approximately 34 g in body weight, which was significantly higher compared to mice fed the CD (mean body weight: 23 g). Obesity results in impaired glucose tolerance and increased glucose levels.^38^ To confirm diet-induced obesity, we measured serum glucose concentrations in both lean and obese mice. Our analysis of mouse serum revealed a clear and statistically significant elevation of glucose levels in obese mice compared to their lean counterparts (Figure S1A). The B cell activating factor (BAFF) has been found to be overexpressed in obesity, leading to an increased abundance of certain B cell subsets.^39^^,^^40^ Besides a strong replication of FV in erythroblasts and myeloid cells, FV also replicates in B cells.^41^^,^^42^ To exclude an obesity-mediated effect on FV target cells, we determined whether the HFD had direct effects on erythroid precursor cells, myeloid cells, B cells within the spleens (Figure S2). Interestingly, absolute numbers of FV target cells in the spleen of HFD-fed mice were similar to CD-fed mice. As acute FV infection leads to mild splenomegaly, we next determined the spleen weight of mice fed with CD or HFD. We did not detect any statistically significant difference between naive mice of both groups (Figure 1C). As expected, spleen weights from both groups significantly increased after FV infection in comparison to naive mice. Interestingly, there was a statistically significant increase in spleen weight in infected mice fed with HFD (mean 0.289 g) compared to CD (mean 0.202 g). Taken together, HFD resulted in diet-induced obesity and augmented splenomegaly after FV infection.

### Decreased NK cell functions in obese mice during acute FV infection

**Figure 2:**
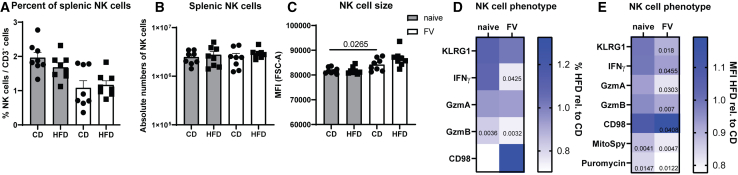
Decreased function of NK cells in obese mice upon acute retrovirus infection For 13 weeks, C57BL/6 mice were fed with HFD or CD. At week 12, mice were infected i.v. with FV for 7 days or used as uninfected, naive control. Splenic NK cells (NK1.1^+^ CD3^−^) were analyzed as percentage (A) or absolute cell numbers (B). NK cells of naive mice are shown in gray, whereas NK cells from FV-infected mice are displayed in white. Statistically significant differences were analyzed by an unpaired t test. Data are presented as mean values ± SEM. FSC-A of NK cells from lean and obese mice are shown in (C). Heat maps show fold changes of percentages (D) and median fluorescence intensity (MFI) (E) of NK cells from naive or FV-infected mice fed with HFD relative to CD (HFD rel. to CD). Eight mice per group from two independent experiments were analyzed by an unpaired t test (KLRG1, CD98, IFNγ, GzmB %, MitoSpy, and puromycin) and Mann-Whitney test (GzmA and GzmB MFI). Statistically significant differences (*p* ≤ 0.05) are indicated in the figure. See also Figure S3.

NK cell functions, such as cytokine production as well as cytotoxicity, are important for the control of viral infections.^27^^,^^43^ We first analyzed the percentages and absolute cell numbers of NK cells in naive and FV-infected animals fed with either CD or HFD (Figures 2A and 2B). We did not detect any statistically significant differences in the frequency or number of NK cells between mice exposed to CD or HFD. NK cell size was determined by analyzing the FSC-A of NK cells, but showed no statistically significant differences between groups of mice fed with CD or HFD (Figure 2C). Instead, we detected an increased NK cell size during FV infection compared NK cells from naive animals in line with previously published data.^13^ Next, we examined the percentages (Figures 2D and S3) and expression (Figures 2E and S3) of KLRG1, a marker associated with NK cell activation and maturation, the pro-inflammatory cytokine IFNγ, and the cytotoxic molecules GzmA and GzmB in NK cells (Figures 2D and 2E). We found a significantly reduced expression of KLRG1 (median fluorescence intensity [MFI]) as well as IFNγ (%, MFI) in obese mice during acute retrovirus infection (Figures 2D and 2E). Cytolytic capacity of NK cells, as shown by GzmA (MFI) and GzmB (%, MFI), was significantly decreased in obese mice infected with FV (Figures 2D and 2E), indicating functional deficits in NK cells. NK cells have to reprogram their metabolism to provide building blocks and energy for activation and cytotoxicity. Interestingly, CD98, a subunit of heterodimeric amino acid transporters, was increased on NK cells from FV-infected animals fed with HFD (Figure 2E, MFI). Mitochondria are important for the generation of ATP, providing energy for NK cells. We observed decreased expression of fluorogenic mitochondria labeling reagent MitoSpy on NK cells from obese mice (Figure 2E), which suggests a decrease in mitochondrial mass. In 2020, Argüello et al. developed a flow cytometry-based method to energetically characterize immune cells by correlating the protein translation (highly energetic process, measured by puromycin incorporation) with ATP concentrations.^44^ Thus, we analyzed the energetic phenotype of NK cells by analyzing puromycin incorporation into NK cells (Figure 2E).^44^ We detected a reduced puromycin incorporation into NK cells from mice fed with HFD, which argues that these cells have decreased ATP availability. These results demonstrate impaired NK cell functions and a diminished energy metabolism in FV-infected, obese mice.

### Decreased cytotoxicity of NK cells in obese mice

**Figure 3:**
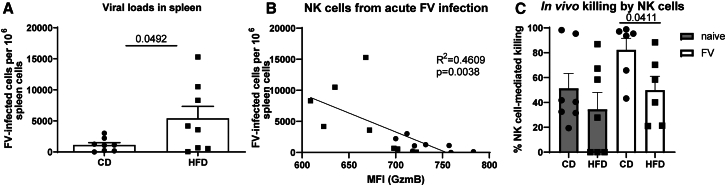
Impaired cytotoxicity of NK cells in obese, retrovirus-infected mice C57BL/6 mice were fed for 13 weeks with HFD or CD. At week 12, some mice were infected with FV for 7 days. Viral loads were analyzed in splenocytes by an infectious center assay (A). Statistically significant differences were analyzed by an unpaired t test. Viral loads were correlated with GzmB expression and goodness of fit and *p* value is displayed (B). Eight mice from at least two independent experiments were used for analyses (A and B). In (C), 5 days post FV infection, mice were injected i.p. with cell tracer-labeled RMA-S tumor cells. CD8^+^ T cells were depleted one day before tumor cell injection. Control animals were depleted for NK cells to calculate the actual killing of NK cells by analyzing the numbers of tumor cells in NK cell-depleted and non-depleted mice ($\frac{\text{Target}\;\text{cells}\;\text{from}\;\text{NK}\;\text{cell}-\text{depleted}\;\text{mice}-\text{Sample}\;\text{target}\;\text{cell}\;\text{number}}{\text{Target}\;\text{cells}\;\text{from}\;\text{NK}\;\text{cell}-\text{depleted}\;\text{mice}}\times 100$). Peritoneal lavage was performed 48 h after tumor cell injection. Seven mice (naive) and six mice (FV) per group from two independent experiments were used and analyzed by Mann-Whitney test. Data are presented as mean values ± SEM. Circles represent individual mice fed with CD, and squares display parameters of obese mice. The significance threshold was set at 0.05.

To understand whether the deficits of NK cell functions (Figure 2) in obesity have an impact on target cell killing *in vivo*, we analyzed the viral loads in CD- and HFD-fed mice at 7 dpi (Figure 3A). FV-infected obese mice had significantly higher viral loads compared to infected mice fed with CD. A strong inverse correlation of the expression of the cytotoxic parameter GzmB in NK cells with viral loads was observed (Figure 3B). This phase of acute FV infection (7 dpi) is characterized by a strong NK cell response^13^ and low T cell activity.^28^ Nevertheless, we analyzed percentages and absolute T cell numbers in the spleen as well as Gzm expression of T cells but did not observe any statistically significant differences between the CD- and HFD-fed group (Figure S4). To demonstrate that the increase in viral burden in high-fat diet-fed mice was connected to decreased NK cell cytotoxicity, an *in vivo* killing assay was performed. Therefore, tumor target cells were injected into the peritoneum of lean and obese mice and analyzed for their cytotoxic potential. The killing capacity of peritoneal NK cells was significantly decreased in FV-infected, obese mice (HFD) in comparison to FV-infected mice fed with CD (Figure 3C). To investigate the potential absence of antiviral functions in NK cells during obesity, we selectively depleted NK cells in an HFD-fed, FV-infected mice. This was achieved through repeated administrations of the NK cell-specific depleting monoclonal antibody PK136 (anti-NK1.1) in mice maintained on HFD. No statistically significant differences in viral loads were observed between NK cell competent and NK cell-depleted mice, which indicate that NK cells do not have an antiviral activity in HFD-rich animals (Figure S5). Collectively, these data show the obesity-induced impairment of NK cell cytotoxic activity.

### A suppressive microenvironment influences NK cell function in obese mice

**Figure 4:**
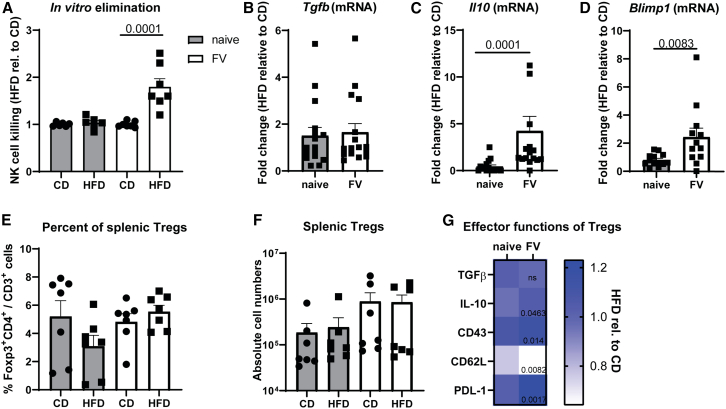
Increased activity of Tregs in HFD-fed mice infected with FV Mice were fed for 13 weeks with HFD or CD. At week 12, some mice were infected with FV for 7 days. Splenic NK cells were isolated using MojoSort Mouse NK cell isolation kit. NK cells were co-cultured with CFSE-stained YAC-1 cells for 4 h and killing was analyzed at BD Canto II (A). Per group, six mice (naive) and seven mice (FV) from at least two independent experiments were used for analyses. Statistically significant differences were analyzed by an unpaired t test (FV, naive). mRNA levels of *Tgfβ* (B), *Il10* (C), and *Blimp1* (D) were analyzed in splenocytes of HFD-mice and normalized to CD. Samples were run in duplets. Eight mice (B and C) and six mice (D) per group from two independent experiments were used and analyzed by Mann-Whitney test. One outlier was removed using ROUT method (D, FV). Percentages (E) and absolute numbers of Foxp3^+^ CD4^+^ Tregs (F) were measured with BD Symphony and analyzed by unpaired t test. Data are presented as mean values ± SEM. Effector functions of Tregs from obese mice, such as TGF-β (Lap-1, MFI), IL-10 (MFI), CD43 (MFI), CD62L (%) and PD-L1 (MFI) were analyzed and normalized to CD-fed mice in G (HFD rel. to CD). Per group, seven mice from two independent experiments were used for analyses (E–G). Statistically significant differences (*p* ≤ 0.05) were analyzed by unpaired t tests and are indicated in the figure. See also Figure S6 ns = not significant.

NK cells traffic and migrate through various microenvironments, thus, they need to adapt to different nutrient and oxygen conditions in order to eliminate target cells. As shown in Figure 3C, the NK cell cytotoxicity *in vivo* was impaired in obese mice. Thus, we addressed the question, whether the impairment of NK cell's cytotoxicity is due to a suppressive environment *in vivo*. Therefore, we isolated NK cells from lean and obese mice and co-cultured them with YAC-1 tumor target cells *in vitro*. If the impairment of NK cell cytotoxicity depends on the suppressive microenvironment, we would expect reinvigorated NK cell killing *in vitro*. As naive NK cells of lean and obese mice displayed only limited alterations in GzmB and GzmA expression (Figures 2D and 2E), we detected no statistically significant differences in the NK cell killing between naive CD- or HFD-groups (Figure 4A). In contrast, an increased killing of target cells was observed, when co-culturing isolated NK cells from FV-infected mice fed with HFD compared to NK cells isolated from lean, infected animals (Figure 4A), demonstrating a strong *in vitro* cytotoxic potential of NK cells isolated from the obese milieu in FV infection. NK cells express various cytokine receptors, and their activity as well as survival is strongly modulated by various cytokines.^45^ Immunosuppressive cytokines such as TGF-β or IL-10 have been demonstrated to influence NK cell metabolism and functions.^30^^,^^46^ Thus, we analyzed *Tgfb* and *Il10* mRNA molecules in splenocytes of naive or FV-infected lean or obese animals (Figures 4B and 4C). We detected comparable *Tgfb* mRNA levels (Figure 4B) but statistically significant differences in *Il10* mRNA molecules (Figure 4C). The level of *Il10* mRNA was strongly increased in a subset of acutely infected obese mice (Figure 4C). The transcription factor Blimp-1 was previously shown to drive the secretion of IL-10 in regulatory T cells (Tregs), thus facilitating obesity and insulin resistance.^47^ Hence, we compared *Blimp1* mRNA in lean and obese mice and observed a statistically significant increase of *Blimp1* mRNA in obese mice infected with FV (Figure 4D). We and others have previously shown that Tregs suppress NK cells through deprivation of IL-2 and secretion of IL-10.^29^^,^^48^ As NK cell cytolytic capacity and NK cell cytokine production was impaired in obese, FV-infected animals (Figure 2D) and the *in vivo* microenvironment might impact NK cell functions (Figures 4C and 4D), we aimed to elucidate whether suppressive Tregs might influence NK cells. Although we neither observed statistically significant differences in the percentage of Tregs (Figure 4E) nor cell numbers (Figure 4F) between FV-infected mice receiving CD or HFD, we observed a significant increase of IL-10^+^ Tregs in obese mice infected with FV (Figures 4G and S6). In line with *Tgfb* mRNA expression data (Figure 4B), we did not detect any statistically significant differences in TGF-β expression in Tregs between CD- and HFD-exposed animals (Figure 4G), indicating that NK cells in obese mice are not suppressed by TGF-β-producing Tregs. However, the flow cytometric analysis of Treg activation revealed an activated phenotype of Tregs as shown for the glycoprotein CD43, L-selectin CD62L and programmed cell death ligand 1 (PD-L1) in obese mice infected with FV (Figures 4G and S6). Taken together, the microenvironment in obesity suppresses NK cells and is probably mediated by IL-10^+^ Tregs.

### The impact of obesity on NK cell cytotoxicity is reversible

**Figure 5:**
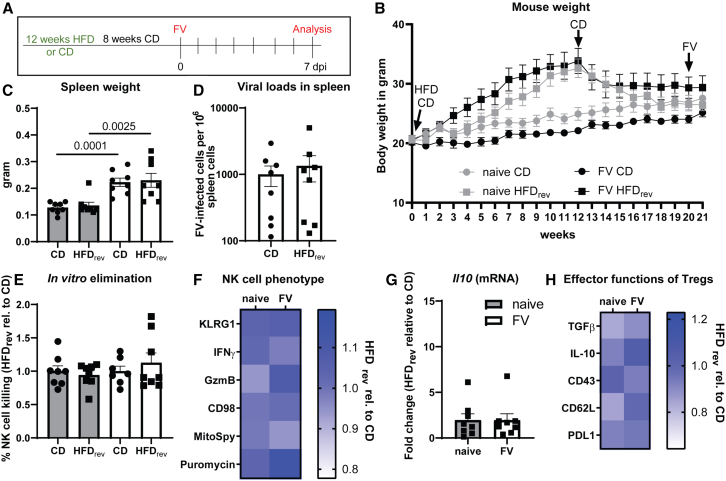
Reinvigoration of NK cell functions in previously obese mice Mice were fed for 12 weeks with HFD or CD. After 12 weeks, all mice were fed with CD for 8 weeks and some mice were infected with FV for 7 days (A). Mouse body weight was measured weekly (B). Spleen weight was determined at day of experiment (C). Statistically significant differences were analyzed by an unpaired t test (CD) and Mann-Whitney test (HFD_rev_). Viral loads were analyzed in splenocytes by an infectious center assay (D). *In vitro* target cell killing of CFSE-labeled YAC-1 tumor cells was detected after 4 h of co-culture (E). Eight mice (naive CD or HFD_rev_, FV HFD_rev_) and seven mice (FV CD) from two independent experiments were analyzed. Data are presented as mean values ± SEM. NK cell functions (MFI) are shown in (F) in a heatmap (HFD rel. to CD). One outlier was removed in MitoSpy analysis by ROUT method (naive, CD). *Il10* mRNA was analyzed in splenocytes of naive and FV-infected CD- and HFD-fed mice and normalized to CD (G). Four mice were used for analysis and samples were run in duplicates. Data are presented as mean values ± SEM. In (H), Tregs were analyzed for TGF-β (MFI), IL-10 (%), CD43 (MFI), CD62L (MFI) and PD-L1 (MFI) (HFD rel. to CD). Eight mice from two independent experiments were used (B and C, F–H). The significance threshold was set at 0.05. rev = reversed.

We described previously that the decreased antiviral NK cell activity in obesity (Figures 2D and 2E) was caused by the suppressive environment (Figures 4C and 4G), potentially through activated Tregs. Thus, we addressed the question whether it is possible to restore NK cell cytotoxicity through weight loss. Mice received CD or HFD for 12 weeks following 8 weeks with CD for both groups (CD, HFD_reversed_ = HFD_rev_). After these 20 weeks, mice were infected with FV or assigned as naive controls (Figure 5A). During the 8 weeks of CD, obese mice lost almost 16% of body weight, which reached its plateau after 4 weeks of CD (Figure 5B). In FV-infected mice, we observed significantly increased spleen weights compared to those of naive mice after 21 weeks but no differences between mice on CD or HFD_rev_ (Figure 5C; white bars). We analyzed the viral loads in FV-infected mice and did not detect any statistically significant differences in ex-obese mice (HFD_rev_) compared to mice fed for 21 weeks with CD (Figure 5D). This indicated that the weight loss in obese mice normalized their ability to control FV infection. We also analyzed the killing capacity of isolated NK cells *in vitro* and observed a similar target cell killing in ex-obese mice (HFD_rev_) compared to CD-fed mice (Figure 5E). Next, we analyzed NK cell functions by analyzing KLRG1, IFNγ, and GzmB as well as the metabolic parameters CD98, MitoSpy, and puromycin expression (Figure 5F). We detected similar expression levels in NK cells of ex-obese mice (HFD_rev_) compared with lean FV-infected mice. Then, we elucidated whether *Il10* mRNA levels and Treg functions were still altered as shown for HFD-only mice (Figures 4C and 4G). We neither detected statistically significant differences in *Il10* mRNA ratios (Figure 5G) nor in the expression of Treg activation markers (Figure 5H) between the groups, indicating that the immunosuppressive milieu in obesity can be reversed by weight loss. Taken together, NK cell activity is reinvigorated and Treg effector function is diminished after reduction of body weight in formerly obese mice, possibly due to reduced activation of immunosuppressive Tregs.

### Impaired antiviral NK cell activity as early as after 10 days of HFD

**Figure 6:**
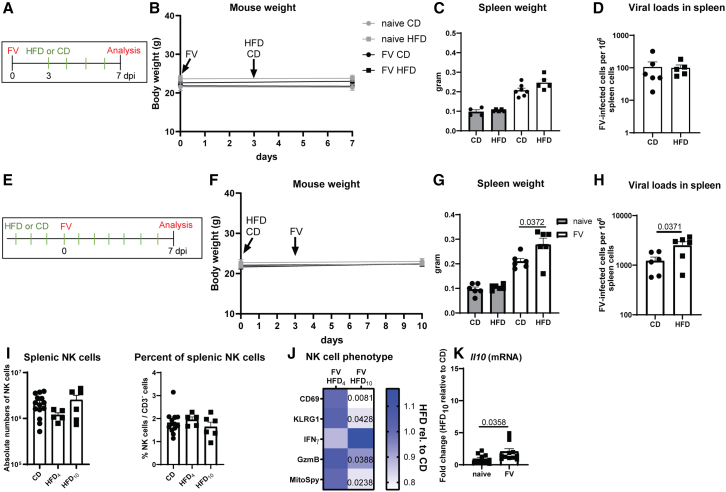
Negative influence of short-term HFD on antiviral NK cells C57BL/6 mice were infected with FV or used as naive controls. After three days, some mice were fed with HFD or left on CD (A). Mice were weighed at the beginning and end of the experiment (B). Spleen weight (C) and viral loads (D) were determined at 7 dpi. Four mice (naive, CD), five mice (naive HFD_4_, FV HFD_4_) and seven mice (FV CD) were used for the analyses and graphs of (B–D). One outlier was removed by applying ROUT method (D; CD). In (E) and (F), mice were fed with HFD or CD for a total of 10 days. After 4 days, mice were infected with FV or left naive. Spleen weight (G) and viral loads (H) were determined at 7 dpi. All groups contain six mice from two independent experiments (F–H). NK cell numbers and percentages are displayed in (I). Outlier analysis was performed using ROUT method (I; numbers, CD). Statistically significant differences were analyzed by an unpaired t test (no significant differences). Heatmap of CD69 (%), KLRG1 (%), IFNγ (MFI), GzmB (MFI) and MitoSpy (MFI) on NK cells (HFD rel. to CD) is shown in (J). At least 4 mice of 4 days and 5 mice of 10 days diet treatment were used for analyses. In (K), *Il10* mRNA levels were determined in duplicates in splenocytes of 10 days CD- (5 mice) or HFD_10_-exposed mice (6 mice). 13 mice (FV, CD), five mice (FV, HFD_4_) and six mice (FV, HFD_10_) from two independent experiments were used for analyses (I, J) and analyzed by an unpaired t test (G, H; CD69, KLRG1, and GzmB of J) or Mann-Whitney test (MitoSpy of J, K). Statistically significant differences (*p* ≤ 0.05) are indicated in the figure. Data are presented as mean values ± SEM.

We have previously shown that the uptake as well as metabolic breakdown of fatty acids through β-oxidation and generation of ATP from fatty acids is important for NK cell migration and subsequent cytotoxic functions.^14^ We therefore hypothesized that short-term treatment with HFD might increase NK cells activity. Therefore, we infected our mice with FV and started the HFD after 3 days for a total period of 4 days (Figure 6A). We measured the body weight, (Figure 6B) spleen weight (Figure 6C), and viral loads (Figure 6D) 4 days post FV infection but did not observe statistically significant differences between CD- or HFD-fed mice. This indicated that 4 days of obesogenic food did not alter the antiviral function of NK cells in FV infection. So, we decided to increase the duration of HFD treatment and fed mice for 3 days with HFD before FV infection (Figure 6E, total of 10 days HFD treatment). Although we did not detect any statistically significant differences in mouse body weights between HFD- and CD-receiving mice (Figure 6F), we observed statistically significantly increased spleen weights after FV infection in mice fed for 10 days with HFD compared to CD (Figure 6G). Interestingly, we already observed increased viral loads after 10 days of HFD compared to CD-fed mice (Figure 6H). We also analyzed the percentage and absolute numbers of NK cells in the spleens of mice fed an HFD for 4 days or 10 days of HFD, and detected comparable levels to the control group (Figure 6I). The analysis of NK cells showed a significantly decreased activation (CD69), maturation (KLRG1), and GzmB expression after feeding mice for 10 days with HFD upon FV infection, but no statistically significant difference in IFNγ production (Figure 6J). Interestingly, MitoSpy was already reduced in NK cells after 10 days of HFD. *Il10* mRNA levels were significantly increased in splenocytes of 10 days HFD-fed mice infected with FV (Figure 6K), suggesting a suppressive microenvironment already after 10 days of HFD. Collectively, these data demonstrate that short-term HFD can have an overall detrimental effect on NK cell functions in a viral infection that involves the disruption of mitochondrial activity and is associated with increases in the inhibitory cytokine IL-10.

### Suppression of antiviral NK cells is mediated by Tregs in mice fed with HFD

**Figure 7:**
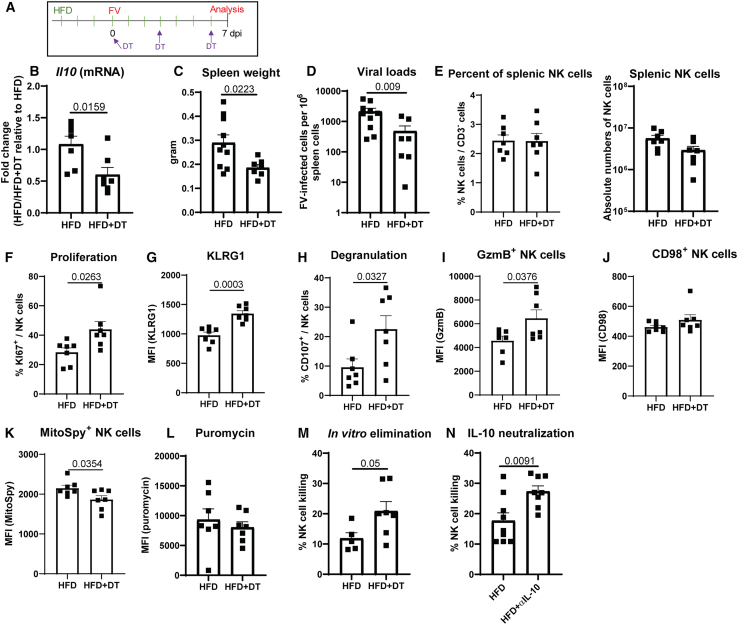
Tregs negatively regulate NK cells after short-term HFD upon acute retrovirus infection DEREG mice were fed with HFD for a total of 10 days. After 4 days, mice were infected with FV for seven days and depleted for Tregs by i.p. injections of DT (A). mRNA of splenocytes was isolated and analyzed for *Il10* expression (B). Seven mice from two independent experiments were used for analyses. Spleen weight (C) and viral loads (D) were determined at 7 dpi (HFD 10 mice, HFD+DT 7 mice). NK cell numbers and percentages are displayed in (E). Proliferation was measured using KI67 (F). Expression of KLRG1 by NK cells is shown in (G). Percentages of degranulating CD107a^+^ NK cells are shown in (H). NK cell cytotoxic capacity was determined by GzmB (I; MFI). Expression levels (MFI) of CD98 (J), MitoSpy (K), and Puromycin (L) in NK cells were analyzed from seven animals of two independent experiments. NK cells were isolated using magnetic beads and co-cultured with CFSE-stained YAC-1 tumor cells. *In vitro* killing was detected with BD Canto II (M). Five mice (HFD) and 7 mice (HFD+DT) from two independent experiments were used to analyze differences between groups. Statistically significant differences were analysed by an unpaired t test (B, C, F–I, K, and M) or Mann-Whitney test (D). IL-10 was neutralized by repetitive injections of anti-IL-10 monoclonal antibodies in short-term HFD mice (N). Splenic NK cells were isolated with magnetic beads and co-cultured with CFSE-stained YAC-1 tumor cells. *In vitro* killing was detected with BD Canto II. Nine mice per group from two independent experiments were used and analyzed by an unpaired t test. One outlier was removed (ROUT method). The significance threshold was set at 0.05. Data are presented as mean values ± SEM. DT: diphtheria toxin.

We demonstrated that Tregs were activated and produced IL-10 in FV-infected obese mice (Figure 4G). Coincidently, NK cells were dysfunctional in these mice. Hence, we hypothesize that Tregs suppress NK cells in obese mice and that their depletion restores NK cell function. To address this, we used DEREG (depletion of regulatory T cells) mice, which carry a diphtheria toxin (DT) receptor-eGFP transgene under the control of the Foxp3 promoter.^49^ This allows the selective depletion of Foxp3^+^ Tregs by application of DT. DEREG mice were fed with HFD for 10 days and were infected with FV after 4 days of HFD. At the same time, Tregs were depleted by 3 repetitive injections of DT (Figure 7A). After 7 days of FV infection, spleens were harvested and mRNA was isolated. We found a significant decrease in *Il10* mRNA in obese mice that were depleted for Tregs compared to obese mice with functional Tregs (Figure 7B). Spleen weight was determined (Figure 7C). We found a statistically significant decrease in spleen weight in DT-treated animals in comparison to mice with Tregs (Figure 7C). Analysis of viral loads revealed a statistically significant reduction in viral burden in Treg-depleted mice compared to non-depleted control mice (Figure 7D). Interestingly, similar NK cell numbers or frequencies of NK cells were detected between both groups (Figure 7E). However, the analysis of NK cell proliferation (Figure 7F), the expression of maturation marker KLRG1 (Figure 7G), degranulation marker CD107a (Figure 7H), as well as GzmB expression (Figure 7I) revealed an augmented proliferation and improved functionality of NK cells in Treg-depleted mice. Interestingly, Treg depletion did not alter the expression of CD98 (Figure 7J) or puromycin levels (Figure 7L) in HFD-fed animals but decreased MitoSpy levels (Figure 7K). Thus, the depletion of Tregs did not result in substantial changes in the metabolic profile of NK cells. Next, we determined the killing capacity of NK cells *in vitro* and found increased target cell killing mediated by NK cells which were isolated from Treg-ablated mice compared to mice with Tregs (Figure 7M). We showed previously that increased levels of *Il10* mRNA as well as higher IL-10 expression by Tregs were found in obese mice. Furthermore, the depletion of Tregs in obese mice resulted in reduced levels of *Il10* mRNA, suggesting that Tregs are the primary source of IL-10 (Figure 7B). To prove that Treg-mediated suppression of NK cells was IL-10-dependent, we neutralized IL-10 in FV-infected mice fed with HFD using monoclonal antibodies. We isolated NK cells and analyzed their cytotoxicity against YAC-1 tumor cells (Figure 7N). We found a statistically significant increase of target cell killing by NK cells isolated from IL-10-neutralized mice. Altogether, NK cell functions were suppressed by IL-10-producing Tregs in mice fed with HFD, suggesting an indirect detrimental effect of extensive lipid exposure on NK cell activity in a viral infection *in vivo*.

## Discussion

In 2022, 2.5 billion adults were living with overweight including over 890 million obese people worldwide (WHO, 2024). These increasing numbers have become a public health and economic problem that leads to the emergence of numerous life-threatening diseases, such as cardiovascular diseases, diabetes, and increased susceptibility to cancer and infections. Infections with viruses cause major health issues worldwide and vaccination as well as curing is still illusive for retroviral infections such as infection with the human immunodeficiency virus (HIV), which infection resulted in approximately 630,000 acquired immunodeficiency syndrome (AIDS)-related deaths in 2022 (UNAIDS, 2024). NK cells are part of the innate immune system and rapidly start to produce proinflammatory cytokines and release cytotoxic granules to induce apoptosis in infected cells but also in transformed cells. Cellular effector functions require energy to fuel increased demands. We and others have demonstrated that the generation of energy from fatty acids, the components of fat, is important for NK cell migration and cytotoxicity in viral infections and cancer.^14^^,^^17^ So, we hypothesized that high levels of free fatty acids, as found in overweight and obesity, increase the NK cell antiviral activity.

The adipose tissue secretes cytokines, called adipokines, such as leptin, adiponectin, or estrogens, which have regulatory functions. It has been shown in some studies that adiponectin as well as estrogens decrease the NK cell cytotoxicity whereas leptin influences the proliferation and migration of NK cells.^50^ Interestingly, we identified similar NK cell numbers in lean and obese FV-infected mice indicating no changes in NK cell proliferation or NK cell motility during obesity.

NK cells are the first line of defense against viral infections, as they do not need prior antigen exposure and they are pre-equipped with cytotoxic molecules. Here, we demonstrate that obesity impairs the cytolytic capacity of splenic NK cells. Decreased GzmB expression was associated with increased viral loads, similar to decreased GzmB expression observed in human NK cells in obesity in the context of cancer.^35^^,^^51^ In addition to decreased cytolytic capacity, splenic NK cells were less able to produce IFNγ in obese mice after FV infection, which is also seen in obese mice challenged with melanoma cells or NK cells from diffuse large B cell lymphoma patients.^35^^,^^51^

In line, we found a decrease in NK cell cytotoxicity in obese mice *in vivo*, when analyzing the NK cell-mediated killing of injected RMA-S cells, a lymphoma cell line devoid of surface major histocompatibility complex (MHC) class I molecules, by peritoneal NK cells. However, after isolation of splenic NK cells from FV-infected obese mice, NK cells were able to eliminate YAC-1 tumor target cells much more efficiently than NK cells from lean mice. YAC-1 cells are fibroblasts isolated from a murine lymphoma. Both cell lines engage distinct pathways of NK cell killing. While NK cells eliminate YAC-1 cells through the recognition of NKG2D ligands and subsequent activation, RMA-S cells become recognized by missing MHC class I, thus decreasing levels of inhibitory signals result in killing of target cells.^52^ Thus, it is tempting to speculate that FV infection in obese mice increase the expression of NKG2D ligands, which results in a higher killing of YAC-1 target cells. Collectively, high levels of free fatty acids might indeed enhance the NK cell cytotoxicity.

There are two main diets that can be used to induce obesity in rodents. A high-fat diet in which mice generate 60% of their calories from fat and a 45% high-fat diet, which we have chosen because it reflects a typical American or European diet (about 36–40% fat by energy) and is more relevant to human physiology.^37^ Jiao et al. found in obese mice (male mice, 60% calories from fat, 5 months on HFD) that the metabolic regulator cMyc was responsible for oleic acid-induced NK cell dysfunction. This dysfunction included defects in cytokine production, GzmB levels, and cytotoxicity.^53^ Obesity was directly linked to metabolic defects in NK cells and decreased cytotoxicity (male mice 60% calories, 8–13 weeks on HFD; female mice 45% calories from fat, 12 weeks on HFD).^35^ Interestingly, we also found reduced frequencies of GzmB^+^ NK cells, a decreased mitochondrial mass and lower energy levels of splenic NK cells in obese mice (45% calories from fat, 13 weeks on HFD). Besides feeding mice with high-fat diets, the lipid metabolism was augmented in a lymphoma environment, which potently inhibited NK cell cytokine production, cytotoxicity, and reduced mitochondrial metabolism.^51^ Another study found that postoperative NK cells upregulated scavenger receptors (CD204, CD36, and CD68), which resulted in augmented lipid accumulation and dysfunctional NK cells.^54^ Consequently, in the context of high-fat diets and conditions characterized by lipid accumulation, excess lipids exert direct inhibitory effects on NK cells.

Interestingly, short-term exposure to obesogenic diet has been shown to render mice susceptible to infectious diseases such as infection with *Listeria monocytogenes*.^55^ In line with this study, retrovirus infection of mice, which were given a high-fat chow for only 10 days, already showed diminished splenic NK cell activity and increased viral burden. Short-term HFD alters intestinal permeability and microbiome composition. It increases gut permeability by stimulating proinflammatory signaling cascades and enhancing barrier-disrupting cytokines.^56^ These changes can occur within 24 h of HFD consumption.^57^ Long-term HFD exacerbates these effects, leading to chronic low-grade inflammation and sustained alterations in gut microbiota. It further compromises gut barrier integrity, reduce the diversity of gut microbiota and affects bile acid composition, which can further damage the intestinal barrier and induce oxidative stress.^57^ It has been reported that several immunosuppressive cell types are linked to obesity, such as myeloid-derived suppressor cells and Tregs,^47^^,^^58^ and we have seen before that Tregs regulate NK cell responses in retrovirus infection.^29^^,^^30^ Interestingly, Tregs are essential for preserving gut barrier integrity and controlling inflammatory immune responses to the microbiota and dietary antigens.^59^ However, Treg numbers and Treg proliferation dramatically decrease in adipose tissues.^60^ In the spleen of FV-infected mice fed with long-term HFD, no statistically significant differences in Treg cell numbers were observed, but Tregs were highly activated and produced the anti-inflammatory cytokine IL-10. In 2021, Beppu et al. report that IL-10 produced by Tregs cause diet-induced obesity.^47^ Interestingly, the transcription factor Blimp-1 was the driver of IL-10 expression in Tregs,^47^ which was also increased in HFD-fed mice infected with FV. In our study, increased levels of *Blimp1* mRNA were detected in murine splenocytes of obese mice and as Blimp-1 is important for plasmablast differentiation, migration, and adhesion, it might reflect *Blimp1* levels in these cells, although B cell numbers were not increased in HFD, and not uniquely in Tregs.^61^ The production of IL-10 by Tregs has been demonstrated to play a crucial role in maintaining intestinal homeostasis. This importance is underscored by the fact that when IL-10 is selectively deleted from Tregs through genetic manipulation, spontaneous colitis develops.^62^ We have shown in short-term HFD that Treg depletion resulted in lower *Il10* mRNA levels in the spleen of FV-infected animals and had a beneficial effect on NK cell responses and subsequently decreased viral loads. Therefore, NK cells are not only directly influenced by high lipid levels, but they are also indirectly regulated by Tregs. However, Treg ablation and IL-10 neutralization strategies require careful consideration of specific contexts, optimal timing, and the choice between local or systemic interventions to maximize therapeutic benefits while minimizing systemic risks and potential side effects. In conclusion, a carefully controlled depletion of Tregs in the context of obesity may enhance NK cell cytotoxicity, potentially leading to a reduced risk of cancer development and improved resistance to viral infections. The analysis of the gut barrier, microbial composition and the absence of Tregs in the context of long-term HFD might be relevant for developing future therapeutic interventions.

For all cellular processes, such as cytotoxicity and cytokine secretion, immune cells require ATP. Only in the first few hours of NK cell activation, NK cells do not require the rearrangement of cellular metabolism.^63^ Michelet et al. showed that NK cells accumulate lipids in obesity, which resulted in an impaired mTOR pathway and loss of function.^35^ Stimulation of NK cells with high concentrations of fatty acids *in vitro* decreased their ATP level.^35^ Interestingly, Koves and colleagues showed that an overload with lipids can impair switching to carbohydrate metabolism in muscle cells.^64^ We identified a strong decrease of mitochondrial mass, which hints to a decrease in mitochondrial OXPHOS, less energetic as well as less cytolytic (GzmB) splenic NK cells in mice fed with HFD.

The regain of physiological condition within the body can be achieved by losing weight through dietary change or physical exercises. Recent reports suggested that physical activity and loss of fat improves NK cells.^65^^,^^66^ Jahn et al. found a significant increase of IFNγ production by NK cells three months after the end of study.^66^ A study by Viel and colleagues demonstrated similar levels of NK cell activation and GzmB expression in ex-obese mice compared to healthy and pre-obese control.^65^ Also, treatment with glucagon-like peptide (GLP-1), which is a gut hormone involved in glucose-dependent insulin secretion and satiety, results in weight loss. Interestingly, GLP-1 therapy resulted in restoration of NK cell metabolism and IFNγ production, which did not correlate with weight loss.^67^ Beside the increased cytokine production and GzmB expression of splenic NK cells in ex-obese mice, we found a comparable killing capacity of NK cells and similar viral loads in comparison to lean FV-infected mice demonstrating the full reinvigoration of NK cell functions in ex-obese mice. Interestingly, suppressive environment in ex-obese mice was also similar to lean, FV-infected animals as shown by IL-10 levels. Moreover, mitochondrial mass and ATP content was comparable in lean and ex-obese groups. In this study, we demonstrate that splenic NK cells are not permanently functionally impaired and weight loss resulted in a regain of NK cell functions.

Similar to a rise of obesity globally, people living with HIV (PLWH) show weight gain and obesity rather than losing weight and wasting, as it was seen in early HIV pandemic. Moreover, obesity in PLWH confers greater risk of metabolic disease in comparison with persons without HIV.^68^ A study with simian immunodeficiency virus (SIV)-infected pigtailed macaques, which gained weight through high-fat chow, showed a worse survival rate of SIV-infected obese compared to SIV-infected lean macaques.^69^ Our findings demonstrate that innate NK cells lose their cytolytic capacity in diet-induced obesity, but regained their cytotoxicity after fat mass reduction. Our findings demonstrate that the obesogenic microenvironment includes activated Tregs and IL-10, both having a suppressive influence on NK cells. Fat mass reduction diminishes the susceptibility to viral infection and opens up new horizons to enhance the NK cell killing of infected cells by specifically modulating the suppressive milieu.

### Limitations of the study

Several limitations of this study should be acknowledged. We used a 45% fat HFD in female mice, which is more physiologically relevant to human diets, instead of the 65% fat HFD commonly used in male mice. This difference may explain limited changes in cytotoxicity and cytokine secretion in naive mice, contrary to previous studies.^35^^,^^53^ However, already short-term HFD significantly altered the NK cell function and increased *Il10* mRNA levels were detected. To show the involvement of Tregs in NK cell dysfunction in HFD, we systemically depleted Tregs in short-term HFD, but function of Tregs in obesity might be different. We analyzed splenic NK cells because FV is highly replicating in this organ; however, NK cell phenotypes in adipose tissue or gut might show more pronounced changes in naive mice on HFD. Finally, our *in vitro* cytotoxicity assays using tumor target cells may not accurately reflect antiviral NK cell activity *in vivo*. These limitations provide opportunities for future research to further clarify the complex relationship between HFD, obesity, and NK cell function in the context of viral infections.

## Resource availability

### Lead contact

Further information and requests for resources and reagents should be directed to and will be fulfilled by the lead contact, Elisabeth Littwitz-Salomon (Elisabeth.Littwitz-Salomon@uk-essen.de).

### Materials availability

This study did not generate new unique reagents.

### Data and code availability

• This study did not generate any large-scale sequencing or proteomics datasets.
• This article does not report original code or source code.
• This study did not generate western blot images or microscopy data.
• Any additional information required to reanalyze the data reported in this paper is available from the lead contact upon request.

## Acknowledgments

L.K. is supported by a grant from 10.13039/100008672Wilhelm Sander Foundation (2023.009.1).

## Author contributions

Conceptualization, E.L.-S.; investigation, S.S., N.K., T.W., L.K., S.a.J., C.C., K.S., and E.L.-S.; analysis, S.S. and E.L.-S.; data discussion, K.S., D.K.F., H.P., and U.D.; writing—original draft, E.L.-S.; writing—review and editing, H.P., K.S., U.D., and E.L.-S.;the final version of the manuscript was reviewed and approved by all authors.

## Declaration of interests

The authors declare no competing interests.

## Declaration of generative AI and AI-assisted technologies in the writing process

The author(s) did not use generative AI technologies for preparation of the publication.

## STAR★Methods

### Key resources table

**Table:** 

REAGENT or RESOURCE	SOURCE	IDENTIFIER
**Antibodies**		

Rat monoclonal anti mouse CD43 (clone S7)	BD Biosciences	Cat# 553271; RRID: AB_394748
Mouse monoclonal anti mouse KI-67 (clone B56)	BD Biosciences	Cat# 564071; RRID: AB_2738577
Rat monoclonal anti mouse CD4 (clone GK1.5)	BD Biosciences	Cat# 565974; RRID: AB_2739427
Mouse monoclonal anti mouse CD69 (H1.2F3)	BioLegend	Cat# 104526; RRID: AB_10679041
Rat monoclonal anti mouse CD98 (4F2) (clone RL388)	BioLegend	Cat# 128216; RRID: AB_2750549
Rat monoclonal anti mouse CD107a (LAMP-1) (clone1D4B)	BioLegend	Cat# 121606; RRID: AB_572007
Mouse monoclonal anti mouse/human GzmB (clone GB11)	BioLegend	Cat# 515408; RRID: AB_2562196
Syrian Hamster monoclonal anti mouse KLRG1 (clone 2F1/KLRG1)	BioLegend	Cat# 138426; RRID: AB_2566554
Mouse monoclonal anti mouse NK1.1 (clone PK136)	BioLegend	Cat# 108732; RRID: AB_2562218
Mouse monoclonal anti mouse LAP (TGF-ß1) (clone TW7-16B4)	BioLegend	Cat# 141404; RRID: AB_10720867
Rat monoclonal anti mouseCD274 (PD-L1) (clone 10F.9G2)	BioLegend	Cat# 124321; RRID: AB_2563635
Armenian Hamster monoclonal anti mouse CD11c (clone N418)	BioLegend	Cat# 117338; RRID: AB_2562016
Rat monoclonal anti mouse CD11b (clone M1/70)	BioLegend	Cat# 101259; RRID: AB_2566568
Rat monoclonal anti mouse F4/80 (clone BM8)	BioLegend	Cat# 123114; RRID: AB_893478
Rat monoclonal anti mouse CD19 (clone 6D5)	BioLegend	Cat# 115561; RRID: AB_2813978
Rat monoclonal anti mouse TER-119 (cloe TER-119)	BioLegend	Cat# 116206; RRID: AB_313707
Rat monoclonal anti mouse Gr-1 (clone RB6-8C5)	Thermo Fisher	Cat# 48-5931-82; RRID: AB_1548788
Rat monoclonal anti mouse FOXP3 (clone NRRF-30)	Thermo Fisher	Cat# 12-4771-82; RRID: AB_529580
Mouse monoclonal anti mouse Gzm A (clone GzA-3G8.5)	Thermo Fisher	Cat# 17-5831-82; RRID: AB_2573228
Rat monoclonal anti mouse IL-10 (clone JES5-16E3)	Thermo Fisher	Cat# 56-7101-82; RRID: AB_891568
Rat monoclonal anti mouse IFNgamma (clone XMG1.2)	Thermo Fisher	Cat# 17-7311-82; RRID: AB_469504
Rat monoclonal anti mouse CD253(TRAIL) (clone N2B2)	Thermo Fisher	Cat# 12-5951-82; RRID: AB_466057
Armenian Hamster monoclonal anti-mouse CD3e (clone 145-2C11)	Thermo Fisher	Cat # 45-0031-82; RRID: AB_1107000
Rat monoclonal anti mouse CD49b (clone DX5)	BioLegend	Cat# 108910; RRID: AB_313417
Mouse monoclonal anti mouse CD62L (clone MEL-14)	BioLegend	Cat# 104428; RRID: AB_830799
Ultra-LEAF Purified anti-mouse IL-10 Antibody (clone JES5-2A5)	BioLegend	Cat# 504909; RRID: AB_2810631
Ultra-LEAF™ Purified anti-mouse NK-1.1 Antibody (clone PK136)	BioLegend	Cat# 108759; RRID: AB_2800569

**Bacterial and virus strains**		

Friend Virus	Institute for Virology, Essen, Prof. Dittmer	N/A

**Critical commercial assays**		

Glucose-Glo Assay	Promega	Cat# J6021
Luna Universal qPCR Master Mix	NEB	M3003LM
eBioscience Foxp3/Transcription Factor Staining Buffer Set	Thermo Fisher	Cat# 00-5523-00
Zombie Aqua Fixable Viability Kit	BioLegend	Cat# 423102
Zombie UV Fixable Viability Kit	BioLegend	Cat# 423108
BD Cytofix/Cytoperm Fixation/Permeabilisation Kit	BD Biosciences	Cat# 554714
MitoSpy Green FM	BioLegend	Cat# 424806

**Experimental models: Cell lines**		

Mouse: M. dunni (fibroblasts)	Institute for Virology, Essen	Clone III8C, CRL-2017
Mouse: YAC-1 (lymphoblasts)	Institute for Virology, Essen	TIB-160
Mouse: RMA-S (mouse leukemia cells)	Institute for Virology, Essen	CVCL_2180

**Experimental models: Organisms/strains**		

Mouse: C57BL/6OlaHsd	Inotiv (Harlan)	Order code 057
Mouse: C57BL/6-Tg(Foxp3-DTR/EGFP)23.2Spar	Institute for Virology, Essen	MGI: 3849080

**Oligonucleotides**		

Primers for TGFb: Mm_Tgfb1_1_SG QuantiTect Primer Assay (QT00145250)	QIAGEN	Cat# 49900
Primers for IL-10: Mm_Tgfb1_1_SG QuantiTect Primer Assay (QT00145250)	QIAGEN	Cat# 249900
Primers for Blimp-1: Forward AAGACGTTCGGTCAGCTCTCCA Reverse CTGGCACTCATGTGGCTTCTCT	Biomers	
Primers for b-Actin: Forward AAATCGTGCGTGACATCAAA Reverse CAAGAAGGAAGGCTGGAAAA	Biomers	

**Software and algorithms**		

GraphPad Prism 8	GraphPad	RRID:SCR_002798
FlowJo V10.8.1	BD	RRID:SCR_008520
Adobe Illustrator 2021	Adobe	RRID:SCR_010279

**Other**		

Control diet	Ssniff	D12450H
High-fat diet	Ssniff	D12451

### Method details

#### Animals

Inbred female C57BL/6J (Harlan Laboratories, Germany) and mixed-sex DEREG mice were at least seven weeks old at the beginning of experiments. Animals were housed in individual ventilated cages under 12:12 light cycle in a relative humidity of 55 ± 10 and a temperature between 22°C ± 2. Mouse experiments were approved from Regional Office for Nature, Environment and Consumer Protection (LANUV, 81-02.04.2021.A458) and performed in strict accordance with the German regulations of the Society for Laboratory Animal Science (GV-SOLAS). Mice were fed *ad libitum* with control diet (Ssniff, similar to Research Diets D12450H) and HFD (Ssniff, lard, similar to Research Diets D12451) for the indicated time.

#### FV infection and infectious center assay

Mice were infected with FV complex containing B-tropic Friend murine leukemia helper virus and polycythemia-inducing spleen focus-forming virus. The FV stock was prepared previously as a 15% spleen cell homogenate from BALB/c mice infected 14 days with 3,000 SFFU of FV. The virus stock was free of lactate dehydrogenase-elevating virus. Mice were injected intravenously with phosphate-buffered saline (PBS) containing 40,000 SFFU of FV and NK cells were analyzed 7 days post infection. Infectious centers were detected by 10-fold dilutions of single-cell suspensions onto *Mus dunni* cells. After 3 days, cells were fixed with ethanol, stained with the F-MuLV envelope-specific monoclonal antibody 720 and developed with a peroxidase-conjugated goat anti-mouse antibody and aminoethylcarbazol to detect foci.

#### Flow cytometry

For flow cytometry stainings the following antibodies were used: CD3 (clone 145.2C11, Thermo) CD4 (GK1.5, BD), CD11b (M1/70, BioLegend), CD11c (N418, BioLegend), CD43 (S7, BD Biosciences) CD49b (DX 5, BioLegend), CD62L (MEL-14, BioLegend), CD69 (H1.2F3, BioLegend), CD98 (4F2, BioLegend), CD107a (ID4B, BioLegend), Foxp3 (NRRF-30, ThermoFisher), GzmA (GzA-3G8.5, Thermo), GzmB (GB11, BioLegend), Gr-1 (RB6-8C5, ThermoFisher), IFNγ (XMG1-2, ThermoFisher), IL-10 (JESS-16E3, ThermoFisher), KI67 (B56, BD Biosciences), KLRG1 (2F1, BioLegend), NK1.1 (PK136, BioLegend), Lap-1 (TW7-16B4, BioLegend), PD-L1 (10F9G2, BioLegend), Ter119 (Ter119, BioLegend), TRAIL (N2B2, ThermoFisher). For exclusion of dead cells, Zombie Aqua™ and Zombie UV™ Fixable Viability Kit (BioLegend) was used. For intracellular cytokine detection, splenocytes underwent *ex vivo* restimulation prior to staining. The cells were incubated for 3 hours at 37°C in RPMI medium supplemented with ionomycin (500 ng/ml), phorbol myristate acetate (PMA) (25 ng/ml), monensin (1X, BioLegend), and brefeldin A (2 μg/ml). Cells were stained with MitoSpy™ Green FM (BioLegend) for 30 min at 37°C (100 nM), washed, surface stained and measured at BD Canto II. Intracellular staining was performed after fixation with BD Cytofix/Cytoperm. For the analysis of Foxp3, cells were fixed with Foxp3/Transcription Factor Staining Buffer Set (Invitrogen). For puromycin staining, RPMI1640 with 10% fetal bovine serum and 100 U/ml Penicillin/100 μg/ml Streptomycin was prewarmed. Puromycin was added (final concentration 10 μg/ml, Sigma) for 20 min. Cells were washed with cold PBS and FC block (BD Biosciences) was performed at 4°C for 5 min. Cell surface was stained at 4°C for 30 min in the dark and cells were washed. Cells were fixed with Fixation/Permeabilization Solution Kit (BD Biosciences). Anti-puromycin (Sigma, clone 12D10) staining was performed in perm-buffer at 4°C for 1 h. Cells were measured at BD Canto II.

#### *In vivo* killing

5 × 10^5^ RMA/S cells were stained with APC Cell Tracer (eBioscience, 80 μM) and injected i.p. One day before and after RMA/S injection, CD8^+^ T cells were depleted with the CD8α-specific depletion antibody (YTS 169.4). Control mice were depleted for NK cells (Leinco, PK136, 20 μg). After 2 days incubation of RMA/S cells in the peritoneum, peritoneal cells were isolated through peritoneal lavage. Cells were measured at BD Canto II. Killing was calculated with following formula: $\frac{\text{Target}\;\text{cells}\;\text{from}\;\text{NK}\;\text{cell}-\text{depleted}\;\text{mice}-\text{Sample}\;\text{target}\;\text{cell}\;\text{number}}{\text{Target}\;\text{cells}\;\text{from}\;\text{NK}\;\text{cell}-\text{depleted}\;\text{mice}}\times 100$.

#### *In vitro* killing

NK cells were isolated with the MojoSort™ mouse NK cell isolation kit (BioLegend) and cocultured with CFSE Cell Division Tracker Kit (BioLegend, 2 μM)-stained YAC-1 cells in a E:T ratio of 25:1. Cells were co-incubated for 4 hours in a humidified 5% CO_2_ atmosphere at 37°C. Cells were washed once and stained with Fixable Viability Dye (ThermoFisher) for dead cell detection. After washing, cells were examined by BD Canto II. Killing was calculated as follows: $\frac{{\text{CFSE}}^{+}{\text{FVD}}^{+}\;}{{\text{CFSE}}^{+}{\text{FVD}}^{-}{+\text{CFSE}}^{+}\text{FVD}+}\times 100$.

#### NK cell depletion and IL-10 neutralization

Mice were injected with 13,3 μg of NK cell depletion antibody (PK136, BioLegend) diluted in PBS. For IL-10 neutralization, 100 μg of Ultra-LEAF™ Purified anti-mouse IL-10 Antibody (JES5-2A5, BioLegend) was injected i.p. Mice were inoculated at day 1, day 3 and day 6 after FV infection.

#### Real-time PCR

Splenocytes were frozen in DNA/RNA Shield (Zymo Research). RNA was extracted using the RNeasy Micro Kit from Qiagen (74004). cDNA was synthesized using the SCRIPT cDNA Synthesis Kit from Jena Bioscience (115482). Real-time PCR was performed in duplicates utilizing the Luna Universal qPCR Master Mix from NEB. QuantiTect Primer assays (Qiagen) for *Tgfb* and *Il10* were used as instructed by the company. As housekeeping gene β-actin was used. The following oligonucleotides were used: *b-actin* 5’-AAATCGTGCGTGACATCAAA-3’, 5’-CAAGAAGGAAGGCTGGAAAA-3; *Blimp1* 5’-AAGACGTTCGGTCAGCTCTCCA-3’, 5’- CTGGCACTCATGTGGCTTCTCT-3; Fold changes were calculated with ΔΔCT method.

### Quantification and statistical analysis

The statistical details of experiments can be found in the figure legends.

Statistics were analyzed and graphs prepared with GraphPad Prism version 8. Statistical differences between two groups were analyzed by an unpaired t test (parametric) or Mann-Whitney test (non-parametric). Differences between multiple groups were examined by Ordinary one-way ANOVA (parametric) and Tukey multiple-comparison test. Linear regression was determined and goodness of fit is displayed in graphs. The significance threshold was set at 0.05. If indicated, outliers were removed using the ROUT method.
