# Supplementary material for: Dietary lipid overload creates a suppressive environment that impedes the antiviral functions of NK cells
Source: iScience. 2025 Apr 9;28(5):112396. doi: 10.1016/j.isci.2025.112396 (PMC12063142; doi:10.1016/j.isci.2025.112396)
Supplement: Document S1. Figures S1–S6 [file mmc1.pdf]

iScience, Volume *28*

Supplemental information

Dietary lipid overload creates a suppressive environment that impedes the antiviral functions of NK cells

Simone Schimmer, Leonie Kerkmann, Nele Kahlert, Shahd al Jubeh, Tanja Werner, Carrie Corkish, Hannah Prendeville, David K. Finlay, Kathrin Sutter, Ulf Dittmer, and Elisabeth Littwitz-Salomon

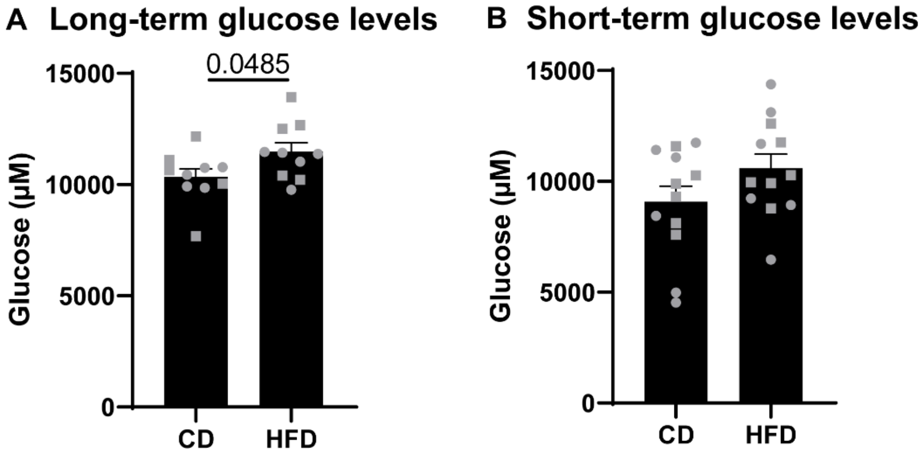

Fig. S1: Glucose levels of CD- and HFD- fed animals. C57BL/6 mice were fed with HFD or CD for a total of 13 weeks (A) or 10 days (B). Mice were infected with FV for 7 days. At 7 dpi, serum was collected and analyzed for glucose (Gucose-Glo Assay, Promega). Circles represent naïve mice whereas squares represent FV-infected animals. Ten mice (A) of one experiment or 12 mice (B) from two independent experiments were analyzed by unpaired t tests. The significance threshold was set at 0.05. Data are presented as mean values ± SEM.

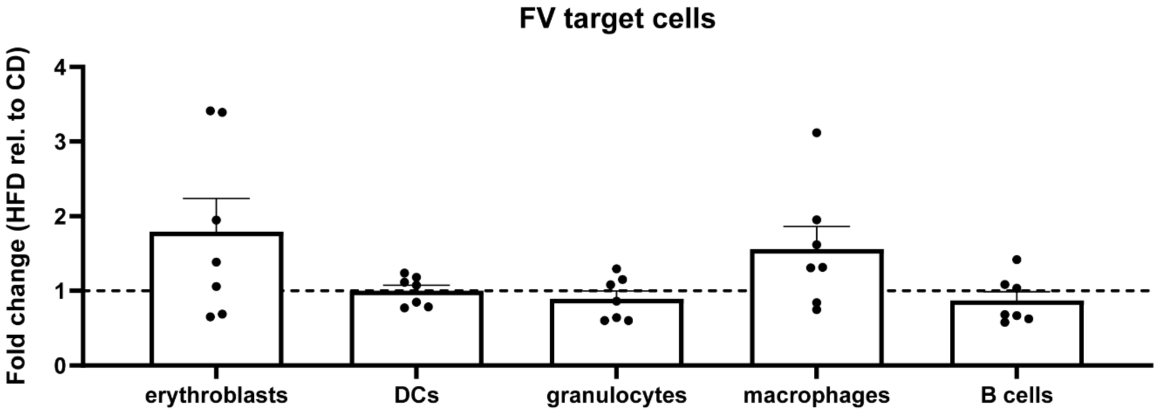

Fig. S2: Influence of short-term diet on FV target cell numbers. C57BL/6 mice were fed with HFD or CD for a total of 10 days. After 4 days, mice were infected with FV. At 7 dpi, spleens were removed and homogenized. Absolute numbers of Ter 119$^+$erythoblasts, CD11c$^+$CD317$^-$ DCs, Gr1$^+$ granulocytes, F4/80$^+$CD11b$^+$ macrophages and CD19$^+$ B cells were analyzed by flow cytometry. Seven mice from two independent experiments were analysed. Data were analyzed by unpaired t tests. The significance threshold was set at 0.05. Data are presented as mean values ± SEM.

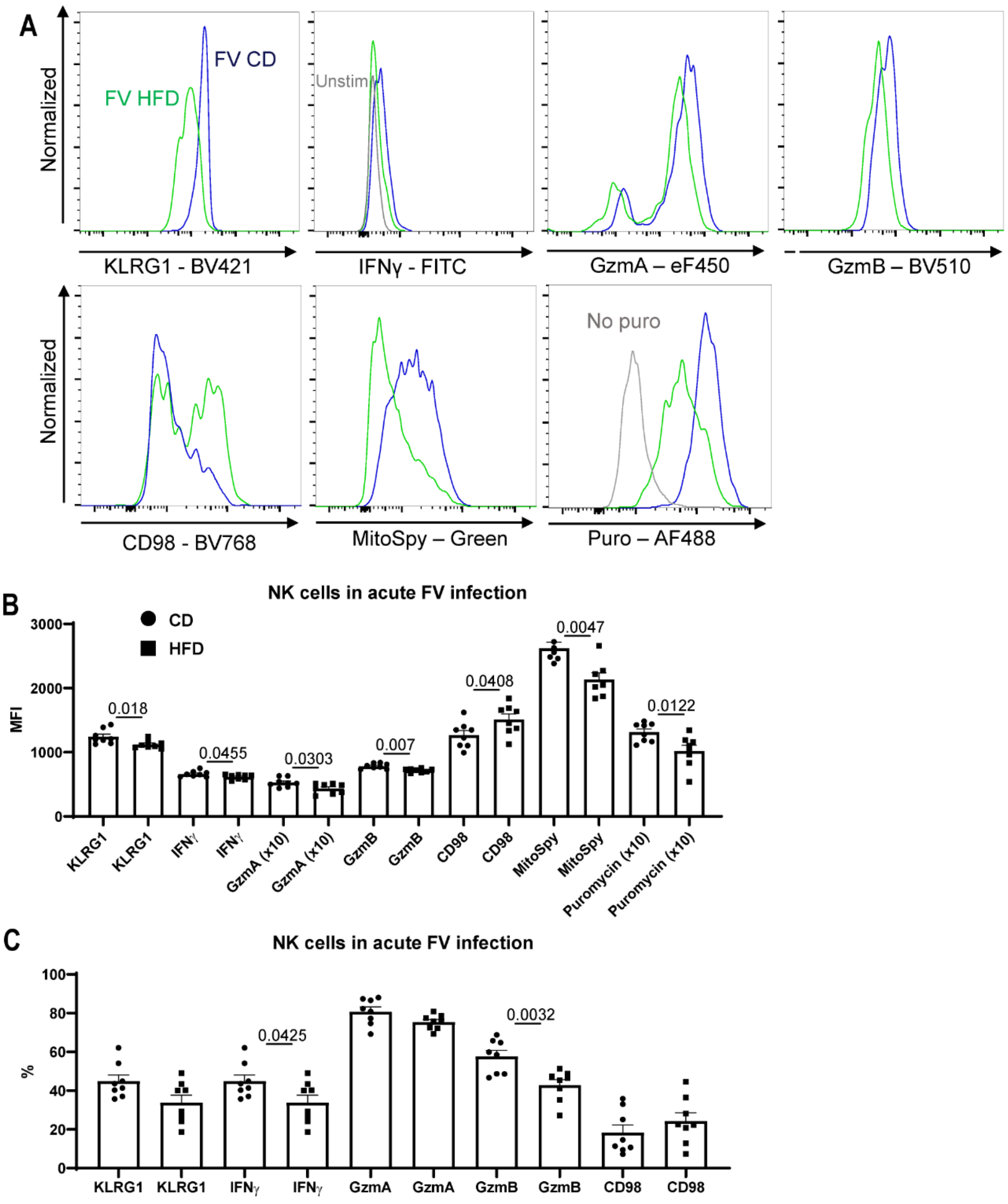

Fig. S3: NK cells phenotype in obese and lean mice in acute FV infection. C57BL/6 mice were fed for 13 weeks with HFD or CD. At week 12, some mice were infected with FV for 7 days. NK cells were analyzed for several parameters, which are displayed as histograms in A. Green line represents NK cells from HFD and blue line from CD. MFI (B) and percentages (C) of KLRG1, IFNγ, GzmA, GzmB, CD98, MitoSpy (MFI), Puromycin (MFI) on NK cells were analyzed by flow cytometry. The significance threshold was set at 0.05. Eight mice per group were analyzed by an unpaired t test (KLRG1, CD98, IFNγ, GzmB %, MitoSpy, Puromycin) and Mann-Whitney test (GzmA, GzmB MFI). Data are presented as mean values ± SEM.

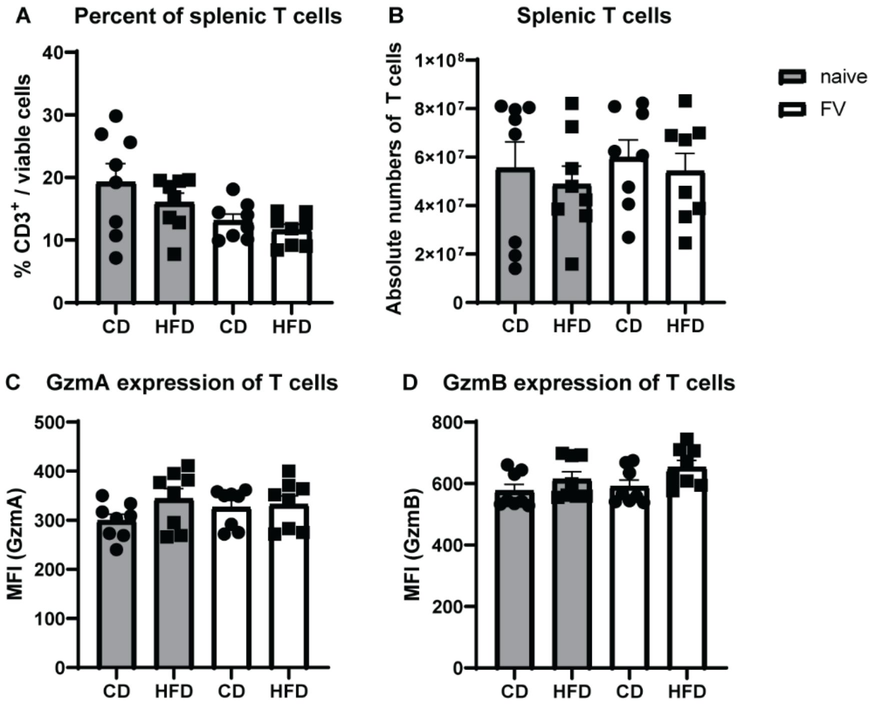

Fig. S4. No influence of HFD on early T cells in acute FV infection. C57BL/6 mice were fed for 13 weeks with HFD or CD. At week 12, some mice were infected with FV for 7 days. The percentage (A) and absolute number (B) of T cells (CD3+) were analyzed by flow cytometry. GzmA (C) and GzmB (D) expression (MFI) was analyzed on T cells. Eight mice from two independent experiments were analyzed. The significance threshold was set at 0.05. Data are presented as mean values ± SEM.

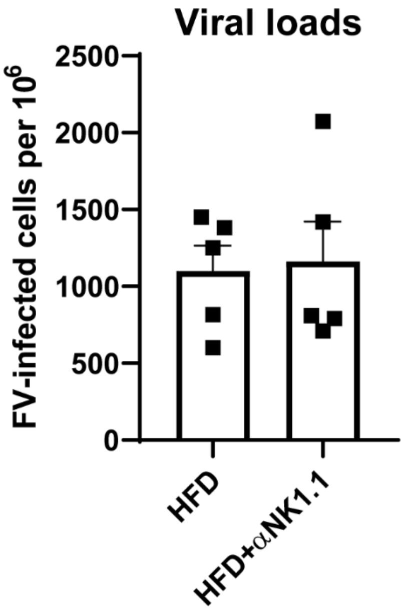

Fig. S5: Viral loads after NK cell depletion in HFD-fed, FV-infected mice. C57BL/6 mice were fed with HFD for a total of 10 days. After 4 days, mice were infected with FV. NK cells were depleted by i.p. injections using a monoclonal antibody (PK136). At 7 dpi, spleens were removed and homogenized. Viral loads were analyzed by infectious center assay. Five mice per group were analysed. Data are presented as mean values ± SEM.

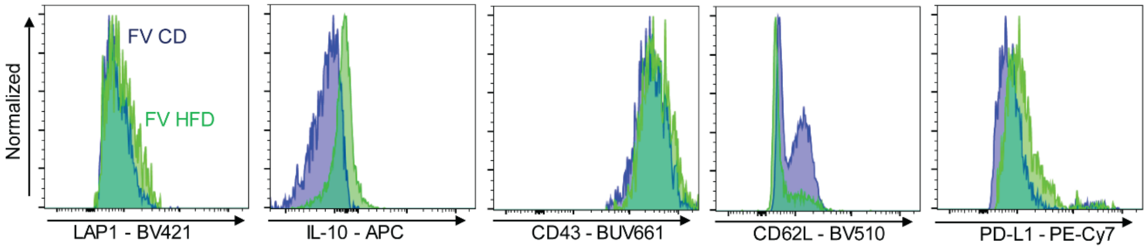

Fig. S6. Function of Tregs in obese, FV-infected mice. C57BL/6 mice were fed for 13 weeks with HFD or CD. At week 12, mice were infected with FV for 7 days. Representative histograms of Tregs TGFβ (LAP1), IL-10, CD43, CD62L and PD-L1 expression from CD- (purple) or HFD-fed (green) animals is shown.
